# Uncertain climate effects of anthropogenic reactive nitrogen

**DOI:** 10.1038/s41586-025-09337-9

**Published:** 2025-10-22

**Authors:** Øivind Hodnebrog, Caroline Jouan, Didier A. Hauglustaine, Fabien Paulot, Susanne E. Bauer, Maureen Beaudor, Michael J. Prather, Marit Sandstad, Ragnhild B. Skeie, Gunnar Myhre

**Affiliations:** 1 Center for International Climate Research (CICERO), Oslo, Norway; 2 Laboratoire des Sciences du Climat et de l’Environnement (LSCE), CEA-CNRS-UVSQ, Université Paris-Saclay, Gif-sur-Yvette, France; 3 Geophysical Fluid Dynamics Laboratory, National Oceanic and Atmospheric Administration, Princeton, NJ USA; 4 NASA Goddard Institute for Space Studies, New York, NY USA; 5 High Meadows Environmental Institute, Princeton University, Princeton, NJ USA; 6 Department of Earth System Science, University of California, Irvine, CA USA

**Keywords:** Atmospheric chemistry, Atmospheric chemistry, Climate and Earth system modelling

arising from: C. Gong et al. *Nature* 10.1038/s41586-024-07714-4 (2024).

The net climate effect of anthropogenic reactive nitrogen (Nr) is the sum of several terms that vary in sign and are associated with substantial uncertainties. Gong et al.^1^ reported a net negative direct radiative forcing (RF) of Nr in the year 2019 relative to the year 1850. We argue that their estimates and associated uncertainties of individual Nr climate effects, most notably aerosol, ozone and methane RF, do not reflect the current state of the art. We show that ref. ^1^ presents overly narrow uncertainty ranges and that their estimates of individual Nr climate effects are outliers compared with our multi-model ensemble, carrying important implications for future projections.

Emissions of Nr lead to the formation of ammonium nitrate aerosols (NH_4_^+^NO_3_^−^; hereafter denoted nitrate), but their atmospheric abundance is highly uncertain. The Intergovernmental Panel on Climate Change Sixth Assessment Report (AR6) states that “there is high confidence that the NH_4_^+^ and NO_3_^−^ burdens have increased from the pre-industrial period to the present day, although the magnitude of the increase is uncertain especially for NO_3_^−^”^2^. The present-day global nitrate burden differs by up to a factor 13 across models in two separate studies^3,4^. This spread holds for fine-mode nitrate aerosols, which drive RF^4^. The complexity of aerosol processes make it challenging to represent nitrate in models. Model diversity in this task has remained almost unchanged between the two latest generations of models^2^.

Sulfate (SO_4_^2−^) aerosols, including ammonium sulfate ((NH_4_^+^)_2_SO_4_^2−^), are also influenced by Nr emissions, mainly through nitrogen oxide (NO_*x*_) emissions, which alter the oxidation pathways of SO_2_ to sulfate by changing the abundances of hydroxyl radicals (OH), ozone (O_3_) and hydrogen peroxide (H_2_O_2_)^5^. Although the latest generation of aerosol-chemistry models are improved, the diversity in modelled sulfate burdens remains considerable^3^ and reproducing observations is still challenging^2,3,6^. Estimates of aerosol RF due to Nr must recognize the large uncertainty reflected in the multi-model intercomparisons.

We have carried out simulations with a set-up similar to ref. ^1^, using five independent latest-generation models (see method description in Supplementary Information), namely, one chemistry-transport model (OsloCTM3 (ref. ^7^)) and four chemistry–climate models (CESM2 (ref. ^8^), GISS ModelE^9^, GFDL-AM4.1 (ref. ^10^) and LMDZ-INCA^11^). The change over the industrial era of nitrate and sulfate aerosol abundances owing to Nr emissions varies greatly across the models, both horizontally (Extended Data Fig. 1a,b) and vertically (Extended Data Fig. 2a,b). Consequently, our estimated direct aerosol RF, which is the RF term with the largest magnitude in ref. ^1^, differs widely by model, even in sign (Fig. 1a and Extended Data Fig. 3a). Our multi-model results show that GEOS-Chem aerosol RF is at the low end (that is, strong cooling). Moreover, none of the other models fall within the GEOS-Chem uncertainty range, which appears to include only emissions uncertainty and not model diversity. The nitrate RF is negative in all models, and the sulfate RF can either add to or counteract the nitrate cooling, depending on the model. The different sulfate RF responses in the models are, at least partly, caused by different responses in the SO_2_ to sulfate oxidants OH and H_2_O_2_ (not shown).

**Fig. 1:**
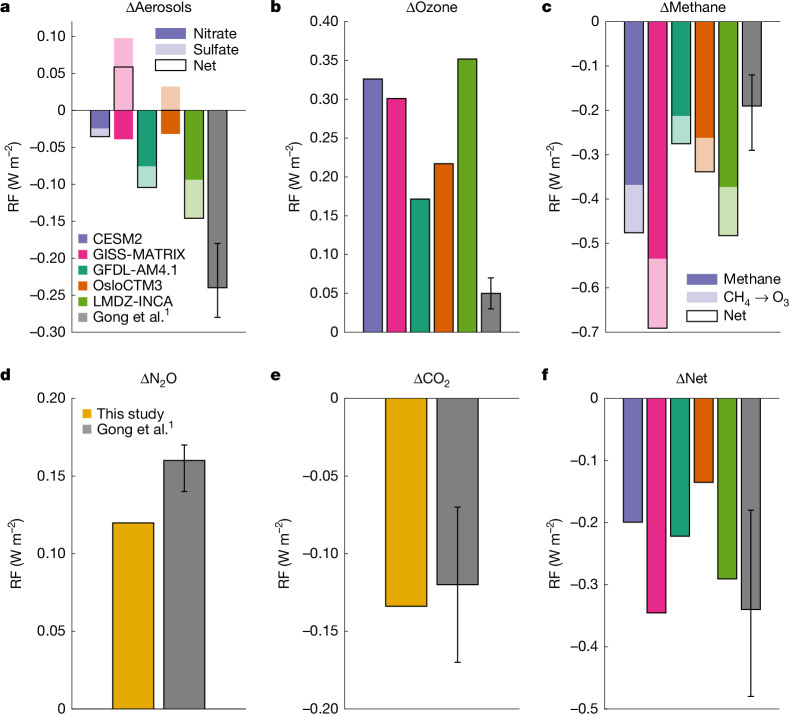
Global pre-industrial to present-day (1850 to 2019) RF due to anthropogenic Nr. Direct aerosol RF (**a**), ozone RF (**b**), methane RF (**c**), N_2_O RF (**d**), CO_2_ RF (**e**) and the net RF calculated as the sum of the individual terms (**f**). The grey bars and whiskers are from ref. ^1^ (see ref. ^1^ for definition of error bars), and the other coloured bars are from this study. N_2_O RF and CO_2_ RF in this study are calculated based on ref. ^14^ and are independent of the model data. RF due to ammonium is included in the nitrate and sulfate terms in **a**.

The RF of ozone due to anthropogenic NO_*x*_ emissions varies widely across models, ranging from 0.07 W m^−2^ to 0.27 W m^−2^ (for 1850 to 2014) in the study used in AR6 (refs. ^2,12^). Here we find a similarly large range in tropospheric ozone caused by anthropogenic Nr emissions (Extended Data Figs. 1c and 2c), and a resulting ozone RF range of 0.17–0.35 W m^−2^ across the five models (Fig. 1b and Extended Data Fig. 3b). These results are a factor 3–7 higher than the GEOS-Chem ozone RF and far outside their reported uncertainty (0.03–0.07 W m^−2^). Although the GEOS-Chem range includes a ±30% uncertainty to account for nonlinear atmospheric chemical reactions, it is applied to their very small ozone RF. The GEOS-Chem results fail to account for the well-known model diversity.

As with ozone, the methane RF due to NO_*x*_ emissions varies considerably across models, partly as a result of differing CH_4_ lifetimes and feedbacks^12^. The common approach of quantifying CH_4_ RF due to NO_*x*_ emissions is to base it on atmospheric chemistry model calculations of CH_4_ lifetime variations due to OH (see Supplementary Information for details). However, ref. ^1^ did not use the GEOS-Chem model for this purpose but rather a CH_4_ box model, which does not properly account for the complex and nonlinear atmospheric chemistry, including effects arising from the inhomogeneous atmospheric distribution of chemical compounds. The well-known effects of CH_4_ being a precursor of tropospheric ozone^13^ and enhancing stratospheric water vapour^2^ have also been ignored. Using our five models and a method in line with AR6 (ref. ^2^), we get a considerably stronger negative CH_4_ RF term than that in ref. ^1^ (Fig. 1c), most of them outside their uncertainty range.

The N_2_O and CO_2_ RF terms due to anthropogenic Nr have been calculated using the RRTMG radiative transfer scheme in GEOS-Chem in ref. ^1^. As these two compounds are well mixed in the atmosphere, and the RRTMG scheme is tailored for fast calculations in global models, we have instead chosen to base the RF calculations on the expressions in ref. ^14^, as in AR6 (ref. ^15^) (see Supplementary Information for details). Assuming the same N_2_O and CO_2_ concentration changes as in ref. ^1^, our calculations give a smaller N_2_O RF term that is outside their uncertainty range (Fig. 1d), but a more similar CO_2_ RF term (Fig. 1e). If tropospheric adjustments would have been added to obtain effective RF (ERF), which is more state of the art, this would change the N_2_O, CO_2_ and CH_4_ forcing by +7 ± 13%, +5 ± 5% and –14 ± 15%, respectively, according to AR6 (ref. ^15^).

Interestingly, the sum of the RF terms gives a net RF that is within the uncertainty range of ref. ^1^ for most models, but with nearly all model estimates being less negative than their net RF (Fig. 1f). Although most of the individual RF terms are very different, our upwards and downwards revisions largely compensate. Although the absolute RF terms can partly cancel, the absolute uncertainty keeps growing as we add the terms. The fact that our individual RF terms differ strongly from those of ref. ^1^ could have large consequences for the future predictions shown in their Fig. 5. We therefore argue that those results cannot be used without applying appropriate uncertainties. We also note that the choice of year for present-day Nr emissions (in this case 2019) could influence the RF results as emissions change rapidly.

Our results emphasize what is clear from previous literature—that a range of models are needed to quantify the climate effects of anthropogenic Nr, including uncertainty. Future research is clearly needed on this important topic, both to better define and narrow the uncertainties on the climate effects given here and (as discussed in ref. ^1^) to quantify climate effects for processes for which estimates do not yet exist (for example, aerosol–cloud interactions due to Nr emissions). Crucially, a natural way forward to reduce uncertainties involves continuous improvement of key processes in the models based on thorough evaluations against a range of observations.

## Supplementary information

Supplementary Information Supplementary Information, including the sections: Global atmospheric chemistry models, Model simulations, Aerosol radiative forcing calculations, Ozone radiative forcing calculations, Methane radiative forcing calculations, N_2_O and CO_2_ radiative forcing calculations, and additional references.

## Figures and Tables

**Extended Data Fig. 1:**
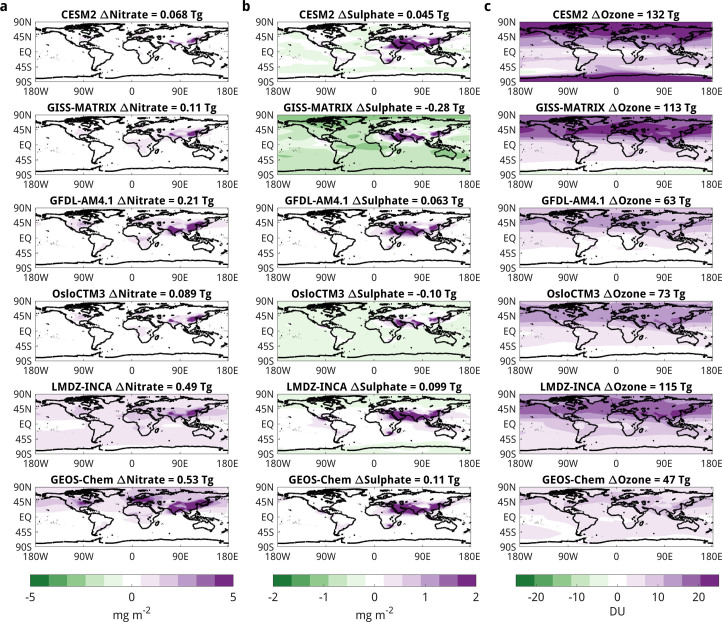
Pre-industrial to present-day (1850 to 2019) change in burden due to anthropogenic Nr. Nitrate fine-mode aerosols (**a**), sulphate aerosols (**b**), and ozone (**c**) for each of the five models in this study and for GEOS-Chem results from ref. ^1^. It is unclear whether the GEOS-Chem results show fine-mode or total nitrate.

**Extended Data Fig. 2:**
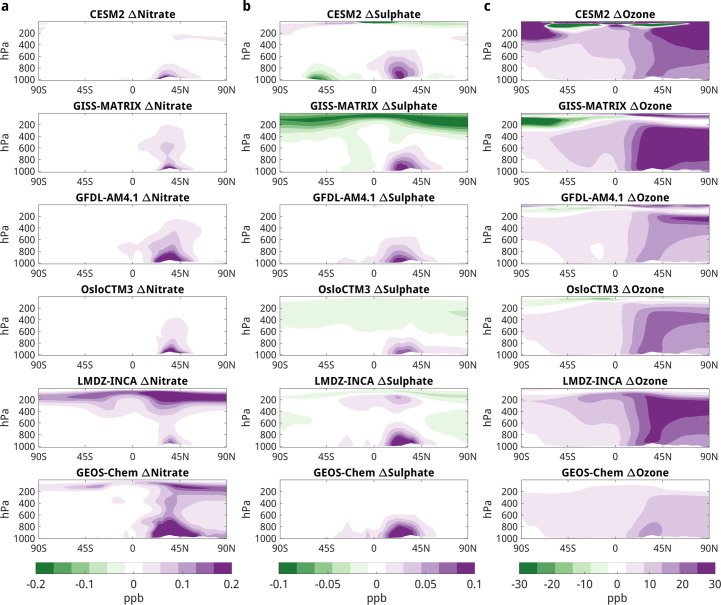
Pre-industrial to present-day (1850 to 2019) zonal mean change due to anthropogenic Nr. Nitrate fine-mode aerosols (**a**), sulphate aerosols (**b**), and ozone (**c**) for each of the five models in this study and for GEOS-Chem results from ref. ^1^. It is unclear whether the GEOS-Chem results show fine-mode or total nitrate.

**Extended Data Fig. 3:**
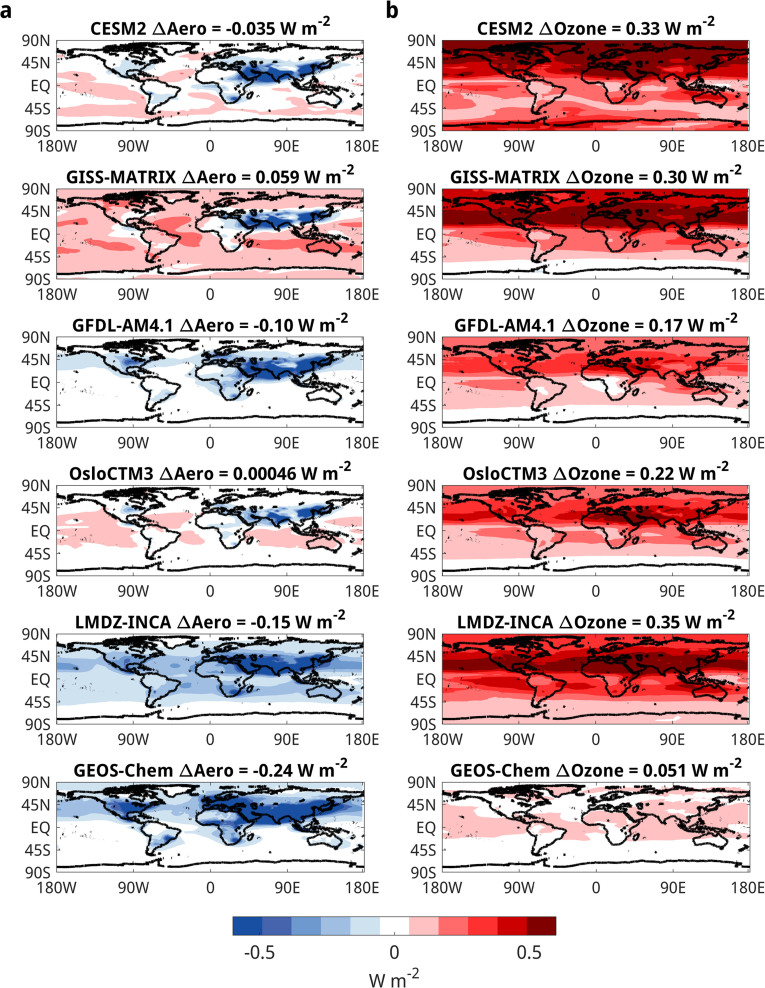
Pre-industrial to present-day (1850 to 2019) radiative forcing (RF) due to anthropogenic Nr. Direct aerosol RF (**a**) and ozone RF (**b**) for each of the five models in this study and for GEOS-Chem results from ref. ^1^. The aerosol RF includes contributions from nitrate, sulphate and ammonium.

## Data Availability

The GEOS-Chem output from Gong et al.^1^ are available on Zenodo at 10.5281/zenodo.11202819 (ref. ^16^). The simulation output from the five models used in this work are available on archive.sigma2.no at 10.11582/2024.00179.
