# Supplementary material for: Uncertain climate effects of anthropogenic reactive nitrogen
Source: Nature. 2025 Oct 22;646(8086):E4–9. doi: 10.1038/s41586-025-09337-9 (PMC12545199; doi:10.1038/s41586-025-09337-9)
Supplement: Supplementary file 1 — Supplementary Information, including the sections: Global atmospheric chemistry models, Model simulations, Aerosol radiative forcing calculations, Ozone radiative forcing calculations, Methane radiative forcing calculations, N2O and CO2 radiative forcing calculations, and additional references. [file 41586_2025_9337_MOESM1_ESM.pdf]

nature portfolio

https://doi.org/10.1038/s41586-025-09337-9

Supplementary information

Uncertain climate effects of anthropogenic reactive nitrogen

In the format provided by the authors and unedited

Supplementary Information

Global atmospheric chemistry models

To simulate climate effects of reactive nitrogen (Nr), defined as all forms of nitrogen except molecular nitrogen (N_2), we have used four chemistry-climate models (CCMs) and one chemistry-transport model (CTM). The five models are very different, both in the sense that they represent CCMs and CTMs and that none are from the same main model family[1].

The Community Earth System Model version 2.2 (CESM2)[2] Community Atmosphere Model version 6 with interactive chemistry (CAM6-Chem)[3] has been run in a version where the four-mode version of the Modal Aerosol Module (MAM4) was coupled to the Model for Simulating Aerosol Interactions and Chemistry (MOSAIC)[4]. This version includes nitrate aerosols and has been run with a horizontal resolution of ~0.9°×1.25° and 32 vertical levels. The model source code is available at https://github.com/ESCOMP/CAM/tree/zz_cam6_3_018_mosaic_branch.

The GISS ModelE version 2.1.2[5] climate model includes interactive gas-phase chemistry and has been run with the Multi-configuration Aerosol Tracker of Mixing State (MATRIX) aerosol module that includes nitrate, using the module EQSAMv3[6], and all other main aerosol compounds. The horizontal resolution is 2°×2.5° and there are 40 vertical layers.

GFDL-AM4.1 is the atmospheric component of the Earth-System Model 4.1[7,8] with a horizontal resolution of ~100 km with 49 vertical levels. Here, we include HNO_3, N_2O_5, NO_3, and SO_2 uptake on dust as described in Paulot et al.[9].

The Oslo Chemistry-Transport Model version 3 (OsloCTM3)[10,11] is driven by three-hourly meteorological forecast data by the Open Integrated Forecast System (Open IFS) at the European Centre for Medium-Range Weather Forecasts, and includes comprehensive chemistry in the troposphere and stratosphere. The horizontal resolution is ~2.25°×2.25° and there are 60 vertical layers. There are 174 chemical gases and aerosols, including all main aerosol compounds such as nitrate[12]. We have used OsloCTM3 v1.1 and the model source code is openly available at https://github.com/NordicESMhub/OsloCTM3.

The LMDZ-INCA global chemistry-aerosol-climate model couples on-line the LMDZ general circulation model (Laboratoire de Météorologie Dynamique)[13] and the INCA model (INteraction with Chemistry and Aerosols)[14-16], and is part of the IPSL Coupled Model[17]. In the present configuration, the model includes 39 hybrid vertical levels extending up to 70 km. The horizontal resolution is 1.25° in latitude and 2.5° in longitude. The model includes a total of 174 tracers, 414 homogeneous and heterogenous chemical reactions, and 80 photolytic reactions.

Model simulations

Each of the models have performed two simulations that have a similar setup as the CTRL_2019 and No_allNr simulations performed with GEOS-Chem in Gong et al.[18].

As in Gong et al., the CTRL_2019 simulation uses 2019 anthropogenic emissions from the Community Emissions Data System (CEDS) version of April 2021, which builds upon the CEDS system described in McDuffie et al.[19]. The only exception is ammonia (NH_3) emissions from the agriculture sector, which we have taken from the CAMEO (Calculation of AMmonia Emissions in ORCHIDEE) dataset of Beaudor et al.[20] as CAMEO gives improved comparison between LMDZ-INCA simulations

and satellite observations[21,22]. CAMEO has also been used for natural ammonia emissions from soil while those from ocean are from Paulot et al.[23] (average of years 2010-2014). Biomass burning emissions for year 2019 are from GFED4.1s[24]. The No_allNr simulation uses the exact same setup as CTRL_2019, except that anthropogenic emissions of NH_3 and NO_X for 2019 were replaced with 1850 emissions from CEDS. In OsloCTM3, global total emissions (anthropogenic, biomass burning and natural) of NH_3 and NO_X in CTRL_2019 are 67.8 Tg N yr^{-1} and 55.8 Tg N yr^{-1}, respectively. The difference in emissions between CTRL_2019 and No_allNr are 45.9 Tg N yr^{-1} and 36.7 Tg N yr^{-1} for NH_3 and NO_X, respectively. These numbers can be compared with 41.3 Tg N yr^{-1} and 36.9 Tg N yr^{-1}, respectively, in Gong et al.[18] (based on their Extended Data Table 1). Although the NH_3 emission perturbation is somewhat higher in our simulations compared to the perturbation in Gong et al.[18], this would not affect our main conclusions.

OsloCTM3 is run for 1 year (2019), as for GEOS-Chem in Gong et al.[18], but uses 1 year of spin-up. The CCMs use nudged meteorology (winds nudged to MERRA-2 in CESM2 and GISS-MATRIX, to NCEP in GFDL-AM4.1[25], and to ECMWF ERA5 in LMDZ-INCA) and need longer simulations to obtain a robust response due to internal variability. The CESM2, GISS-MATRIX and GFDL-AM4.1 model simulations ran for 20 meteorological years of 2000-2019, but with emissions being the same for each year, and the last 10 years were analyzed. In LMDZ-INCA, the aerosols and ozone fields calculated and perturbed did not interact with radiation. This provided a much better signal and it was sufficient to run LMDZ-INCA for 1 year (2019) as for OsloCTM3.

Aerosol radiative forcing calculations

In LMDZ-INCA, the aerosol radiative forcing (RF) is calculated online in the simulations (see refs.[15,16] for RF model description). For the other models, and for the spatial distribution plot for LMDZ-INCA (in Extended Data Fig. 3), the aerosol RF of ammonium nitrate and ammonium sulphate are calculated using the monthly mean 3D distributions of nitrate (in fine-mode) and sulphate, respectively, from each model simulation combined with a 3D aerosol kernel that gives RF per mass (W g^{-1})[26] (available at https://github.com/ciceroOslo/Radiative-kernels). It should be noted that the forcing calculated using the kernel approach would not match exactly the forcing that would be calculated interactively, especially in GISS-MATRIX where aerosols are internally mixed. The aerosol kernel was generated based on perturbations of sulphate aerosols, and this purely scattering kernel is here used for nitrate aerosols, but we have accounted for differences in molecular weight. Lund et al.[11] showed that the normalized RF (W g^{-1}) was very similar between sulphate and nitrate (see their Table S5). To be consistent with Gong et al.[18], we report the direct aerosol effect as the difference in all-sky RF at the top-of-atmosphere (TOA) between the CTRL_2019 and No_allNr simulations. In AR6 terminology, this would be RF of aerosol-radiation interaction (RFari) and, as adjustments following aerosol-radiation interactions for purely scattering aerosols are negligible[27], it can be assumed the same as effective RF (ERFari). It should be noted that the RF of aerosol-cloud interaction (RFaci; indirect aerosol effect) and its adjustments (ERFaci) are not accounted for here or in Gong et al.[18], and could have a large impact on the aerosol climate effect[28].

Ozone radiative forcing calculations

To calculate ozone RF we have used the monthly mean 3D ozone distribution from each model simulation combined with a 3D ozone kernel that gives RF per dobson unit (DU) for each of the shortwave and longwave components[29] (available at https://github.com/ciceroOslo/Radiative-kernels). The ozone RF are for all-sky at TOA and includes stratospheric temperature adjustment but not tropospheric adjustments. AR6 assessed that the ERF was equal to the RF that includes stratospheric temperature adjustment, because of insufficient evidence for quantification of adjustments due to ozone[28].

Methane radiative forcing calculations

Methane is relatively well-mixed in the atmosphere due to its long lifetime, and the surface concentration of methane is therefore held fixed in all model simulations. A common approach is to quantify methane RF caused by changes in ozone precursor emissions based on the OH-induced changes in methane lifetime (e.g.[30-33]). In each of the CTRL_2019 and No_allNr simulations we calculate the methane lifetime due to OH by the reaction CH_4 + OH, based on the monthly mean 3D distributions of the two gases. The lifetime of methane due to OH is then combined with a lifetime of 240 years due to loss in the stratosphere (adjusted from 120 years to avoid double-counting of loss due to OH[34]) and 160 years due to soil deposition[30] to obtain the total methane lifetime in each simulation. The change in methane concentration (ΔC) caused by the difference in methane lifetime between the two simulations is then estimated (see e.g., ref.[35]) by

$$\Delta C = f \times C_{2019} \times \frac{\Delta\tau}{\tau_{\mathrm{No\_allNr}}}$$

where f = 1.30 is the feedback factor to account for the impact of methane changes on its own lifetime[33], C_{2019} = 1866.3 ppb is the 2019 methane mixing ratio[28], $\tau_{\mathrm{No\_allNr}}$ is the total methane lifetime in the No_allNr simulation, and $\Delta\tau$ is the difference in total methane lifetime between the CTRL_2019 and No_allNr simulations. The methane concentration change (ΔC) is then multiplied by a radiative forcing efficiency to obtain the methane RF. This radiative forcing efficiency is calculated for a 1 ppb change from C_{2019} based on the expressions in Etminan et al.[36] to be 0.438 mW m^{-2} ppb^{-1} and we have added 0.04 mW m^{-2} ppb^{-1} to account for methane-induced changes in stratospheric water vapor[28] and 0.14 mW m^{-2} ppb^{-1} to account for methane-induced ozone[28]. The calculated methane RF are for all-sky at TOA and includes stratospheric temperature adjustment but not tropospheric adjustments. If tropospheric adjustments would have been added to obtain ERF, this would change the CH_4 forcing by –14±15% according to AR6[28].

N_2O and CO_2 radiative forcing calculations

The N_2O and CO_2 RF are calculated using the expressions in Etminan et al.[36], which are based on detailed line-by-line radiative transfer calculations. This is the same method as used in AR6, except that the Meinshausen et al.[37] expressions that were re-fitted from Etminan et al.[36] were used in AR6. We assume the same concentrations of N_2O, CO_2 and CH_4 for the No_allNr and CTRL_2019 experiments as in Gong et al.[18] (their Extended Data Table 1). The calculated N_2O and CO_2 RF are for all-sky at TOA and includes stratospheric temperature adjustment but not tropospheric adjustments. If tropospheric adjustments would have been added to obtain ERF, this would increase the N_2O and CO_2 forcing by +7±13% and +5±5%, respectively, according to AR6[28].

References

1 Kuma, P., Bender, F. A.-M. & Jönsson, A. R. Climate Model Code Genealogy and Its Relation to Climate Feedbacks and Sensitivity. *Journal of Advances in Modeling Earth Systems* **15**, e2022MS003588, doi:https://doi.org/10.1029/2022MS003588 (2023).

2 Danabasoglu, G. *et al.* The Community Earth System Model Version 2 (CESM2). *Journal of Advances in Modeling Earth Systems* **12**, 35, doi:10.1029/2019ms001916 (2020).

3 Emmons, L. K. *et al.* The Chemistry Mechanism in the Community Earth System Model Version 2 (CESM2). *Journal of Advances in Modeling Earth Systems* **12**, doi:10.1029/2019ms001882 (2020).

4 Lu, Z. *et al.* Radiative Forcing of Nitrate Aerosols From 1975 to 2010 as Simulated by MOSAIC Module in CESM2-MAM4. *Journal of Geophysical Research: Atmospheres* **126**, e2021JD034809, doi:https://doi.org/10.1029/2021JD034809 (2021).

5 Bauer, S. E. *et al.* Historical (1850-2014) Aerosol Evolution and Role on Climate Forcing Using the GISS ModelE2.1 Contribution to CMIP6. *Journal of Advances in Modeling Earth Systems* **12**, doi:10.1029/2019ms001978 (2020).

6 Metzger, S., Dentener, F., Pandis, S. & Lelieveld, J. Gas/aerosol partitioning: 1. A computationally efficient model. *J. Geophys. Res.-Atmos.* **107**, doi:10.1029/2001jd001102 (2002).

7 Dunne, J. P. *et al.* The GFDL Earth System Model Version 4.1 (GFDL-ESM 4.1): Overall Coupled Model Description and Simulation Characteristics. *Journal of Advances in Modeling Earth Systems* **12**, e2019MS002015, doi:https://doi.org/10.1029/2019MS002015 (2020).

8 Horowitz, L. W. *et al.* The GFDL Global Atmospheric Chemistry-Climate Model AM4.1: Model Description and Simulation Characteristics. *Journal of Advances in Modeling Earth Systems* **12**, e2019MS002032, doi:https://doi.org/10.1029/2019MS002032 (2020).

9 Paulot, F. *et al.* Sensitivity of nitrate aerosols to ammonia emissions and to nitrate chemistry: implications for present and future nitrate optical depth. *Atmos. Chem. Phys.* **16**, 1459-1477, doi:10.5194/acp-16-1459-2016 (2016).

10 Søvde, O. A. *et al.* The chemical transport model Oslo CTM3. *Geosci. Model Dev.* **5**, 1441-1469, doi:10.5194/gmd-5-1441-2012 (2012).

11 Lund, M. T. *et al.* Concentrations and radiative forcing of anthropogenic aerosols from 1750 to 2014 simulated with the Oslo CTM3 and CEDS emission inventory. *Geosci. Model Dev.* **11**, 4909-4931, doi:10.5194/gmd-11-4909-2018 (2018).

12 Myhre, G., Grini, A. & Metzger, S. Modelling of nitrate and ammonium-containing aerosols in presence of sea salt. *Atmos. Chem. Phys.* **6**, 4809-4821, doi:10.5194/acp-6-4809-2006 (2006).

13 Hourdin, F. *et al.* LMDZ6A: The Atmospheric Component of the IPSL Climate Model With Improved and Better Tuned Physics. *Journal of Advances in Modeling Earth Systems* **12**, e2019MS001892, doi:https://doi.org/10.1029/2019MS001892 (2020).

14 Hauglustaine, D. A. *et al.* Interactive chemistry in the Laboratoire de Meteorologie Dynamique general circulation model: Description and background tropospheric chemistry evaluation. *J. Geophys. Res.-Atmos.* **109**, doi:10.1029/2003jd003957 (2004).

15 Hauglustaine, D. A., Balkanski, Y. & Schulz, M. A global model simulation of present and future nitrate aerosols and their direct radiative forcing of climate. *Atmospheric Chemistry and Physics* **14**, 11031-11063, doi:10.5194/acp-14-11031-2014 (2014).

16 Terrenoire, E. *et al.* Impact of present and future aircraft NOx and aerosol emissions on atmospheric composition and associated direct radiative forcing of climate. *Atmos. Chem. Phys.* **22**, 11987-12023, doi:10.5194/acp-22-11987-2022 (2022).

17 Boucher, O. *et al.* Presentation and Evaluation of the IPSL-CM6A-LR Climate Model. *Journal of Advances in Modeling Earth Systems* **12**, e2019MS002010, doi:https://doi.org/10.1029/2019MS002010 (2020).

18 Gong, C. *et al.* Global net climate effects of anthropogenic reactive nitrogen. *Nature* **632**, 557-563, doi:10.1038/s41586-024-07714-4 (2024).

19 McDuffie, E. E. *et al.* A global anthropogenic emission inventory of atmospheric pollutants from sector- and fuel-specific sources (1970–2017): an application of the Community Emissions Data System (CEDS). *Earth Syst. Sci. Data* **12**, 3413-3442, doi:10.5194/essd-12-3413-2020 (2020).

20 Beaudor, M., Vuichard, N., Lathière, J. & Hauglustaine, D. Global ammonia emissions from CAMEO throughout the century for 3 scenarios (2000-2100) (Version 2) [Data set]. https://doi.org/10.5281/zenodo.8324858. *Zenodo* (2023).

21 Beaudor, M. *et al.* Global agricultural ammonia emissions simulated with the ORCHIDEE land surface model. *Geosci. Model Dev.* **16**, 1053-1081, doi:10.5194/gmd-16-1053-2023 (2023).

22 Beaudor, M. *et al.* Evaluating present-day and future impacts of agricultural ammonia emissions on atmospheric chemistry and climate. *Accepted for publication in Atmos. Chem. Phys.* **2024**, 1-40, doi:10.5194/egusphere-2024-2022 (2024).

23 Paulot, F., Stock, C., John, J. G., Zadeh, N. & Horowitz, L. W. Ocean Ammonia Outgassing: Modulation by CO2 and Anthropogenic Nitrogen Deposition. *Journal of Advances in Modeling Earth Systems* **12**, e2019MS002026, doi:https://doi.org/10.1029/2019MS002026 (2020).

24 van der Werf, G. R. *et al.* Global fire emissions estimates during 1997–2016. *Earth Syst. Sci. Data* **9**, 697-720, doi:10.5194/essd-9-697-2017 (2017).

25 Lin, M. *et al.* Transport of Asian ozone pollution into surface air over the western United States in spring. *Journal of Geophysical Research: Atmospheres* **117**, doi:https://doi.org/10.1029/2011JD016961 (2012).

26 Samset, B. H. & Myhre, G. Vertical dependence of black carbon, sulphate and biomass burning aerosol radiative forcing. *Geophys. Res. Lett.* **38**, doi:10.1029/2011gl049697 (2011).

27 Stjern, C. W. *et al.* The Timescales of Climate Responses to Carbon Dioxide and Aerosols. *J. Clim.*, 1-28, doi:https://doi.org/10.1175/JCLI-D-22-0513.1 (2023).

28 Forster, P. M. *et al.* in *Climate Change 2021: The Physical Science Basis. Contribution of Working Group I to the Sixth Assessment Report of the Intergovernmental Panel on Climate Change* (eds V. Masson-Delmotte *et al.*) 923–1054 (Cambridge University Press, 2021).

29 Skeie, R. B. *et al.* Historical total ozone radiative forcing derived from CMIP6 simulations. *Npj Climate and Atmospheric Science* **3**, 10, doi:10.1038/s41612-020-00131-0 (2020).

30 Stevenson, D. S. *et al.* Tropospheric ozone changes, radiative forcing and attribution to emissions in the Atmospheric Chemistry and Climate Model Intercomparison Project (ACCMIP). *Atmos. Chem. Phys.* **13**, 3063-3085, doi:10.5194/acp-13-3063-2013 (2013).

31 Bellouin, N. *et al.* Regional and seasonal radiative forcing by perturbations to aerosol and ozone precursor emissions. *Atmospheric Chemistry and Physics* **16**, 13885-13910, doi:10.5194/acp-16-13885-2016 (2016).

32 Thornhill, G. D. *et al.* Effective radiative forcing from emissions of reactive gases and aerosols - a multi-model comparison. *Atmospheric Chemistry and Physics* **21**, 853-874, doi:10.5194/acp-21-853-2021 (2021).

33 Szopa, S. *et al.* in *Climate Change 2021: The Physical Science Basis. Contribution of Working Group I to the Sixth Assessment Report of the Intergovernmental Panel on Climate Change* (eds V. Masson-Delmotte *et al.*) 817–922 (Cambridge University Press, 2021).

34 Sand, M. *et al.* A multi-model assessment of the Global Warming Potential of hydrogen. *Communications Earth & Environment* **4**, 203, doi:10.1038/s43247-023-00857-8 (2023).

35 Berntsen, T. K. *et al.* Response of climate to regional emissions of ozone precursors: sensitivities and warming potentials. *Tellus Ser. B-Chem. Phys. Meteorol.* **57**, 283-304 (2005).

36 Etminan, M., Myhre, G., Highwood, E. J. & Shine, K. P. Radiative forcing of carbon dioxide, methane, and nitrous oxide: A significant revision of the methane radiative forcing. *Geophys. Res. Lett.* **43**, 12614-12623, doi:10.1002/2016gl071930 (2016).

37 Meinshausen, M. *et al.* The shared socio-economic pathway (SSP) greenhouse gas concentrations and their extensions to 2500. *Geosci. Model Dev.* **13**, 3571-3605, doi:10.5194/gmd-13-3571-2020 (2020).
